# Rationale and design of ePPOP-ID: a multicenter randomized controlled trial using an electronic-personalized program for obesity in pregnancy to improve delivery

**DOI:** 10.1186/s12884-020-03288-x

**Published:** 2020-10-07

**Authors:** Philippe Deruelle, Sophie Lelorain, Sylvie Deghilage, Emmanuelle Couturier, Elodie Guilbert, Paul Berveiller, Marie Victoire Sénat, Christophe Vayssière, Loïc Sentilhes, Franck Perrotin, Denis Gallot, Céline Chauleur, Nicolas Sananes, Emmanuel Roth, Dominique Luton, Marie Caputo, Elodie Lorio, Carla Chatelet, Julien Couster, Oumar Timbely, Muriel Doret-Dion, Alain Duhamel, Marie Pigeyre

**Affiliations:** 1 Univ. Lille, CHU Lille, EA 4489 - Environnement Périnatal et Santé, F-59000 Lille, France; 2 Pôle Gynécologie, Obstétrique et Fertilité, Hôpitaux Universitaires de Strasbourg, 67200 Strasbourg cedex, France; 3 Univ. Lille, CNRS, CHU Lille, UMR 9193 – SCALab – Cognitive and Affective Sciences, Lille, France; 4 Department of Obstetrics and Gynecology, Poissy Saint Germain hospital, Poissy, France; 5 Assistance Publique-Hôpitaux de Paris, Department of Gynecology-Obstetrics, Bicêtre Hospital, University of Paris-Sud, University of Medicine Paris- Saclay Le Kremlin-Bicêtre, Le Kremlin-Bicêtre, France; 6 Department of Obstetrics and Gynecology, Paule de Viguier Hospital, CHU Toulouse, Toulouse, France; 7 Department of Obstetrics and Gynecology, Bordeaux University Hospital, Bordeaux, France; 8 Department of Gynecology and Obstetrics, Inserm U1253 « Imaging and Brain » (iBrain). CHU Bretonneau, Tours, France; 9 Pôle Femme Et Enfant, CHU Estaing, Clermont-Ferrand cedex 1, France; 10 R2D2-EA7281, Université d’Auvergne, Faculté de Médecine, Place Henri Dunant, Clermont-Ferrand, France; 11 INSERM, SAINBIOSE, U1059, Dysfonction Vasculaire et Hémostase, Université Jean-Monnet, CIC1408, F- 42055 Saint-Etienne, France; 12 Maternal Fetal Medicine Department, INSERM 1121 “Biomaterials and Bioengineering”, Strasbourg University Hospital, Strasbourg, France; 13 Department of Obstetrics and Gynecology, AP-HP, Bichat hospital, Paris, France; 14 Department of Obstetrics and Gynecology, Lens general hospital, Lens, France; 15 Department of Obstetrics and Gynecology, Valenciennes general hospital, Valenciennes, France; 16 Department of Obstetrics and Gynecology, Béthune general hospital, Béthune, France; 17 Department of Obstetrics and Gynecology, Boulogne general hospital, Boulogne, France; 18 Department of Obstetrics and Gynecology, Meaux general hospital, Meaux, France; 19 Department of obstetrics and gynecology surgery, Femme mere enfant university hospital, hospices civils de Lyon, Bron, France; 20 Univ. Lille, CHU Lille, EA 2694 - Santé publique : épidémiologie et qualité des soins, F-59000 Lille, France; 21 Department of medicine, endocrinology division, Mc Master university, Hamilton, Canada

**Keywords:** Obesity, Pregnancy, Cesarean delivery, Instrumental delivery, Physical activity, Nutrition, Fetus, Macrosomia

## Abstract

**Background:**

Pre-pregnancy obesity and excessive gestational weight gain (GWG) are established risk factors for adverse pregnancy, delivery and birth outcomes. Pregnancy is an ideal moment for nutritional interventions in order to establish healthier lifestyle behaviors in women at high risk of obstetric and neonatal complications.

**Methods:**

Electronic-Personalized Program for Obesity during Pregnancy to Improve Delivery (ePPOP-ID) is an open multicenter randomized controlled trial which will assess the efficacy of an e-health web-based platform offering a personalized lifestyle program to obese pregnant women in order to reduce the rate of labor procedures and delivery interventions in comparison to standard care. A total of 860 eligible pregnant women will be recruited in 18 centers in France between 12 and 22 weeks of gestation, randomized into the intervention or the control arm and followed until 10 weeks of postpartum.

The intervention is based on nutrition, eating behavior, physical activity, motivation and well-being advices in which personalization is central, as well as the use of a mobile/tablet application. Inputs includes data from the medical record of participants (medical history, anthropometric data), from the web platform (questionnaires on dietary habits, eating behavior, physical activity and motivation in both groups), and adherence to the program (time of connection for the intervention group only). Data are collected at inclusion, 32 weeks, delivery and 10 weeks postpartum. As primary outcome, we will use a composite endpoint score of obstetrical interventions during labor and delivery, defined as caesarean section and instrumental delivery (forceps and vacuum extractor). Secondary outcomes will consist of data routinely collected as part of usual antenatal and perinatal care, such as GWG, hypertension, preeclampsia, as well as fetal and neonatal outcomes including premature birth, gestational age at birth, birth weight, macrosomia, Apgar score, arterial umbilical cord pH, neonatal traumatism, hyperbilirubinemia, respiratory distress syndrome, transfer in neonatal intensive care unit, and neonatal adiposity. Post-natal outcomes will be duration of breastfeeding, maternal weight retention and child weight at postnatal visit.

**Discussion:**

The findings of the ePPOP-ID trial will help design e-health intervention program for obese women in pregnancy.

**Trial registration:**

ClinicalTrials.gov Identifier: NCT02924636 / October 5th 2016.

## Background

Obesity is a major public health threat and has been listed as the sixth most important risk factor contributing to the overall burden of disease worldwide. In 2016, the World Health Organization estimated that there were 1.9 billion individuals with a body mass index (BMI) > 25 kg/m^2^ including nearly 325 million obese women (BMI > 30 kg/m^2^) [1]. Obesity affects approximately 15% of French population, involves more frequently young women [2] and represents a significant and increasing problem encountered in obstetrics [3]. There are well documented risks associated with obesity in pregnancy including gestational diabetes mellitus (GDM), hypertensive disorders and preeclampsia, thromboembolic diseases, stillbirth but also difficulties during delivery such as caesarean section, instrumental extraction related to macrosomia and sources of neonatal trauma. Following delivery, obese women are more likely to suffer from post-partum hemorrhage and to have longer hospital stays than women with a normal BMI (18.5–24.9 kg/m^2^) [3]. The effects of obesity may extend beyond health in pregnancy, as increasing evidence suggests that children of obese mothers or of those with excessive gestational weight gain (GWG) may be at greater risk of obesity because of exposure to adverse metabolic influences in utero, or in the early postnatal period [4]. Excessive GWG also increases the risk of maternal and neonatal complications. Excessive GWG, especially if superimposed on pre-existing excess of weight, increases the risk of obstetric complications including gestational hypertension, GDM, preeclampsia, and caesarean delivery in mothers; and macrosomia and long-term obesity in offspring [4]. In addition, excessive GWG would worsen maternal obesity in a long-term fashion, as excessive GWG is a major determinant of postpartum weight retention [5, 6].

The Institute of Medicine (IOM) published GWG guidelines according to the pre-pregnancy BMI in order to improve maternal as well as fetal outcomes. For a BMI between 20 and 25 kg/m^2^, recommended GWG should be between 11 and 16 kg, whereas for a BMI over 30 kg/m^2^, the GWG should be between 5 and 9 kg, in order to decrease the risk of adverse pregnancy outcomes. These recommendations are based on observational studies suggesting more favorable outcomes with lower GWG [7]. However, the United Kingdom National Institute for Health and Clinical Excellence guidelines on dietary and physical activity interventions for weight management before, during and after pregnancy, concluded that more evidence of outcome improvements from interventional studies is required before these or similar guidelines for limitation of GWG would be adopted [8].

In a systematic review of nine randomized trials including 743 overweight and obese pregnant women, there was no significant effect of interventions designed to limit GWG on weight gain or on delivery of a large-for-gestational-age (LGA) infant [9] and another systematic review of four trials addressing dietary interventions to decrease GWG, reported a reduction in GWG among 537 obese pregnant women without any influence on birth weight [10].

A recent systematic review of interventions in overweight and obese pregnant women observed that overall interventions were associated with reduced GWG, but without evidence for any effect in birth weight or caesarean section rates; although the available studies were considered of poor to medium quality [11]. A meta-analysis of 10 trials on GWG in normal, overweight and obese pregnant women, concluded that dietary advices during pregnancy appear effective in decreasing GWG and long term postpartum weight retention, but evidence for benefits on infant and maternal health was limited [12]. A second systematic review on GWG suggested that physical activity may reduce GWG with little evidence for improved outcomes [13]. In a third systematic review of 12 trials in normal BMI and obese pregnant women (*n* = 1656 women), dietary and physical activity interventions were effective in reducing GWG, but there was considerable heterogeneity in outcomes [14]. The analysis highlighted differences in sample characteristics and aspects of intervention design, content, delivery and evaluation, which might explain discrepancies in effectiveness. The most recent meta-analysis included 44 trials and concluded that dietary and lifestyle interventions in pregnancy could reduce GWG and improve outcomes for both mothers and newborns, although the overall evidence level was low to very low for major outcomes such as preeclampsia, GDM, gestational hypertension, and preterm delivery. Only five trials could be used to evaluate the effect of interventions on caesarean section. The authors stated that ongoing effectiveness trials should focus on clinically relevant outcomes such as caesarean section and instrumental delivery [15]. The LIMIT trial indicated that provision of lifestyle advices to women who were overweight or obese during pregnancy did not reduce the risk of large for gestational age (LGA) infants or improve maternal outcomes in pregnancy and birth, but was associated with a significant reduction in risk of macrosomia defined as birthweight superior to 4 kg [16].

In total, the current available data suggest that dietary and physical interventions in pregnancy are effective at reducing the GWG compared to usual care, while they do not increase the risk for small-for-gestational-age (SGA) or low birth weight in newborns.

However, controlling GWG within the recommendations is not easily achievable for many pregnant women, especially those already overweight or obese. Indeed, 30 to 50% of obese women have a GWG beyond the guidelines, even in the groups who follow an antenatal lifestyle program [15]. Although the current IOM guidelines recognize that “interventions will be needed to assist women, particularly those who are in overweight or obese at the time of conception” to meet these recommendations. There is little high-quality evidence available from randomized trials to determine the best approach to manage GWG in pregnancy. Published studies were mainly monocentric with interventions judged to be too complex or too expensive to be implemented widely. There was considerable variation in the nature of the interventions provided in the previously published works, ranging from single session with a dietician to dietetic counselling sessions at each antenatal visit. Although the provision of a more intensive program has been associated with greater weight loss in non-pregnant individuals [17, 18], the ability to provide this type of intervention at a broader antenatal population remains questionable. Moreover, there are barriers to lifestyle modification in pregnant women, including lack of time and energy, competing work and family demands, and lack of childcare [19].

Considering implementation of the web in the society, we hypothesized that using a personalized and pragmatic program combining nutritional, physical activity, motivational and well-being counselling, would address this issue. A pilot study using SMS messaging intervention demonstrated the feasibility, acceptability, and potential efficacy of low-intensity and expendable intervention to help overweight and obese women reduce GWG. Although the results did not reach statistical significance due to small sample size (*n* = 14 in the intervention group and *n* = 9 in the control group), women in the intervention group had a GWG of six pounds less than participants in the control group (95% CI − 15.9, 4.0; *p* = 0.24) at 40 weeks of gestation [20].

The introduction of the new digital technologies in the society increased since the last century. For people, Internet technologies have become so ubiquitous as to seem invisible. The implementation difference of the Internet is low between workers and managers [21], and mobile phone use appears to be similar across all socioeconomic groups [22]. In fact, some socially disadvantaged populations are *more* likely to send messages everyday than their more advantaged counterparts. Providing a personalized prevention program is innovative by the use of technologies that are essential tools for daily life: cell phones, computers or tablets to access the Internet and emails. Thus, the use of tools that become universal could help to sensitize the pregnant women. Moreover, pregnancy is a very privileged time to implement good health habits and to diffuse them within the family. E-mail and newsletter interventions, as well as online forum should be more acceptable for obese pregnant than face-to-face and telephone counselling or group sessions. Women will not feel judged or devalued. An electronic intervention may be especially useful for self-monitoring because of the potential for providing both support and immediate feedback based on a patient’s specific goals. This system will fight against geographical or social disparities. Indeed, the Internet is spread to the entire country regardless of health resources available nearby. The “Institut National de Statistiques” considers that only 3% of the French citizens do not have the possibility to access to the Internet [23]. Online interventions have emerged as a popular strategy to promote healthy behaviors. However, the limited amount of work done with Internet-based obesity interventions has provided little in the way of solid and reproducible results. A systematic review of eight Internet-based randomized trials reported some improvement with mixed findings, due to small sample sizes. The authors stated that the research including the use of technologically based interventions should be amplified and targeted to answer specific questions [24].

To our knowledge, there is no published work that studied the effect of a technologically based intervention during the pregnancy. A program was recently tested in the post-partum period. The Diabetes Prevention Program demonstrated that an intensive, face-to-face lifestyle intervention could achieve weight loss and reduce incidence of type 2 diabetes in middle-aged adults at high-risk, including women with a GDM history [25]. However, because face-to-face lifestyle intervention studies in post-partum women have had limited success [25, 26] and given the multiple barriers to face-to-face interventions, and the widespread use of the Internet, the authors adapted the Diabetes Prevention Program into a web-based lifestyle intervention using web-based technology to deliver lifestyle interventions for women with recent GDM. The study demonstrated the feasibility and efficacy of the web-based lifestyle modification program to decrease postpartum weight retention in the first postpartum year for women with recent GDM. The authors concluded that this program is at low cost to implement and has potential for broad dissemination [19]. These data suggest that a web-based program should be tested in obese pregnant women to limit GWG.

The purpose of our study is to assess the efficacy of an e-personalized program including antenatal dietary and lifestyle advices in pregnancy to reduce the rate of labor procedures and interventions in comparison to standard care. As secondary objectives, we will investigate whether the e- program may improve the health of the mothers and their newborns, compared to a control group allocated to standard care. Maternal secondary outcomes include reducing GWG, increasing the proportion of participants having a GWG within the guidelines, reducing the risk of hypertension, preeclampsia, and GDM, reducing fat and sugar intake, increasing physical activity, increasing breastfeeding and duration, reducing weight retention after childbirth. For newborns, secondary outcomes will consist of improving birth weight, reducing risk of macrosomia, trauma associated with childbirth and low Apgar score, reducing the use of phototherapy for hyperbilirubinemia, as well as reducing number of admissions to neonatal intensive care unit and number of infants with excessive weight at postnatal visit.

## Methods

### Study setting

**Fig. 1:**
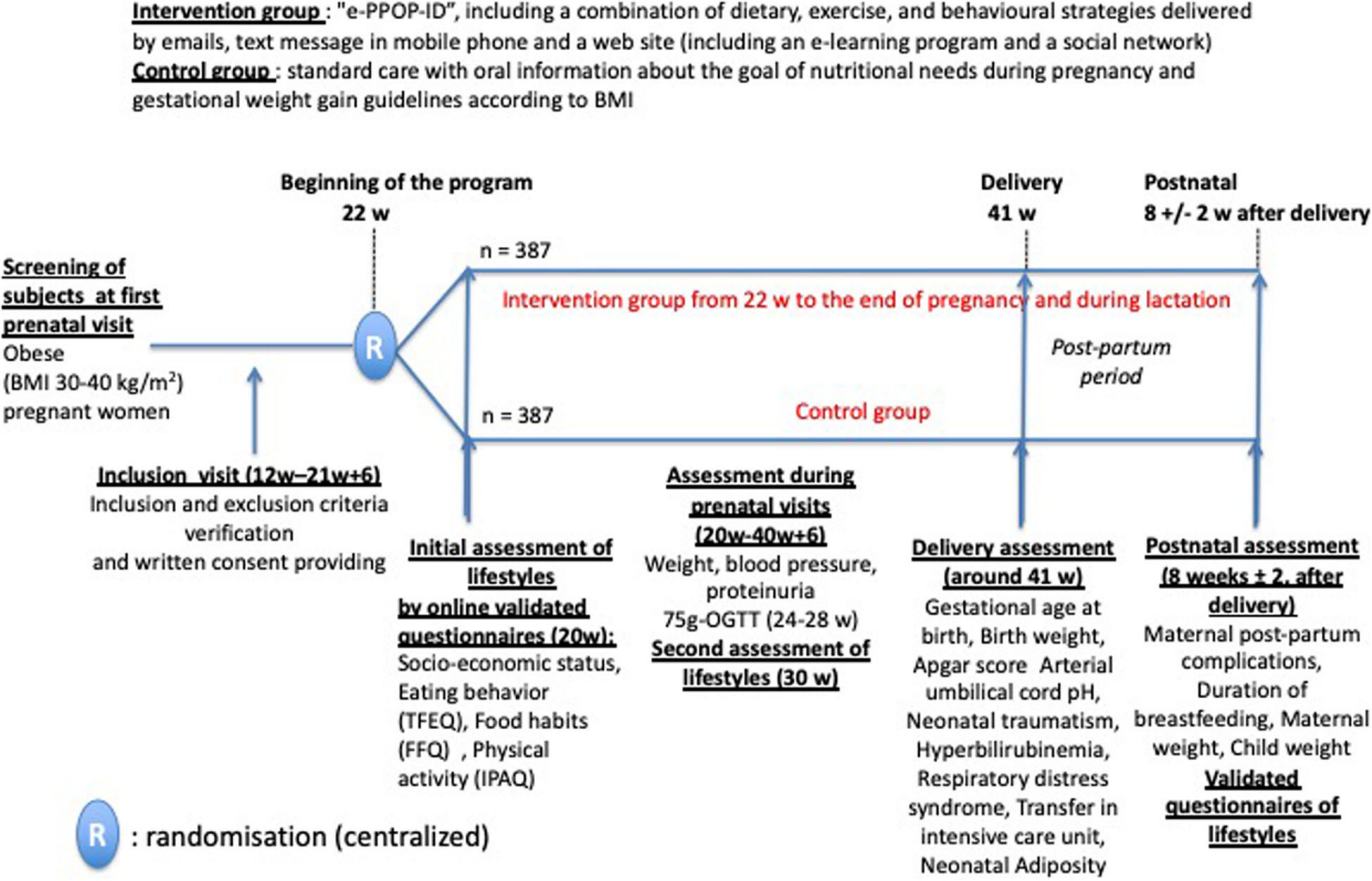
Protocol overview. Electronic-Personalized Program for Obesity during Pregnancy to Improve Delivery (ePPOP-ID) is an open multicenter randomized controlled trial which will assess the efficacy of an e-health web-based platform offering a personalized lifestyle program to obese pregnant women in order to reduce the rate of labor procedures and delivery interventions in comparison to standard care. A total of 860 eligible pregnant women will be recruited in 18 centers in France between 12 and 22 weeks of gestation, randomized into the intervention or the control arm and followed until 10 weeks of postpartum. For both arms, inputs include data from the medical record of participants and the web platform (online questionnaires), collected at inclusion, 32 weeks, delivery and 10 weeks postpartum

ePPOP-ID is an open multicenter randomized controlled trial, which involves 18 university and/or regional hospitals from nine regions in France (study design illustrated in Fig. 1 and Table 1). These centers deliver approximately 30,000 patients per year, which leads to a potential of 2400 to 3000 obese pregnant women per year (i.e. 8 to 10% of the pregnant women being obese), which makes 200 to 250 eligible women per month.

**Table 1:**
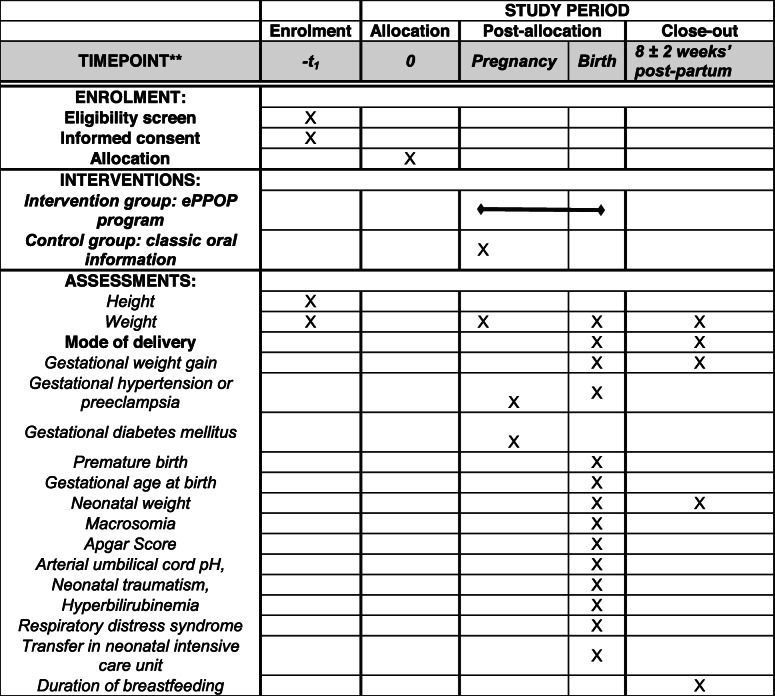
Schedule of enrolment, interventions, and assessments for ePPOP-ID Study

### Recruitment and eligibility criteria

Screening of participants is performed by midwives and obstetricians of involving hospitals at the first prenatal visit, between 12 and 14 weeks of gestation, at which a verification of inclusion and exclusion criteria is anticipated for the inclusion visit.

To optimize the recruitment, the study was presented to all caregivers that perform visits. Flyers and posters are placed in the waiting rooms of the prenatal consultation area in order to inform potential participants.

Inclusion visit takes place between 12 and 22 weeks. Pregnant women with a singleton pregnancy, a pre-pregnancy BMI between 30 and 40 kg/m^2^, and aged of 18–45 years-old are included. Other eligibility criteria consist of providing written informed consent, having access to internet (by using phone, tablet or computer), being comfortable with the use of internet, and having an email address. Exclusion criteria consist of having a history of more than 2 miscarriages, severe heart disease (arrhythmias, history of myocardial infarction), unstable thyroid disease, uncontrolled hypertension, pre-gestational diabetes, bariatric surgery or any medical condition that may interfere with physical activity during pregnancy, no health insurance, or being legally assisted or unable to consent. Participants can not follow other lifestyle interventions during their participation in the ePPOP-ID trial. The total duration of the trial will be 50 months with an enrolment period of 24 months. The participants are withdrawn of the study if they lose the ability to connect to the program.

### Randomization

The randomization procedure is performed at the end of the inclusion visit, centralized and stratified by center. The 1:1 assignment sequence (based on blocks of four and the use of a computer random-number generator) is produced by the sponsor. The randomization list is not disclosed to the study centers, monitors, statisticians or to the trial team. After collection of the email address of participants, an invitation is sent to each participant to connect to the web-based platform, in order to complete the registration procedure (i.e. to complete the profile and define a login password), as well as fill in the online initial assessments. The intervention group also has access to the personalized electronic lifestyle program whereas the control group not. Due to the nature of the intervention a blind study was not achievable.

### Intervention

The intervention is presented as a comprehensive dietary and lifestyle intervention called “e-PPOP” (electronic-Personalized Program for Obesity in Pregnancy), including a combination of diet, exercise, and behavior strategies available on a web-based platform specifically developed by BePatient™ according to our program requirements. The e-health platform is accessible through the web when using computer, or through an application when using tablet and smartphone. The platform includes patient’s and professional’s modules. The patient’s modules have been designed to help obese pregnant women change their lifestyle and improve their health through a personalized e-learning program (Additional file 1). The professional’s modules provide a remote follow-up of patient activities, such as questionnaires completion, self-monitoring of weight, e-learning program adherence. A social network is also available to facilitate contacts between patients and between patients and caregivers.

The individualization of the e-learning program has been essential in the development of our educational module. Personalization follows an algorithm based on participant’ answers to the initial online assessments, covering eating behavior, physical activity, diet and motivation competences (Additional file 2).

#### Patient’s module

A comprehensive e-learning module offers to participants to access a personalized lifestyle program elaborated by our multidisciplinary team, including dietician, psychologist, physical activity coach, midwife and physician. It provides educational contents, which have been elaborated according to the French guidelines for nutrition and physical activity during pregnancy [27] and evidence-based behavior-change counseling [28–31]. The objectives of the e-learning program are to limit GWG by encouraging balanced intake of carbohydrates, fat and protein; reducing high-energy foods intake (i.e. refined carbohydrates and saturated fats); increasing intake of fruits and vegetables; and, also, by encouraging physical activity practice.

The e-learning supports are composed by a total of 162 factsheets (covering behavioral messages, nutritional information on needs during pregnancy and breastfeeding, recipes), videos of exercises and yoga classes, recording of relaxation class, as well as quizzes to evaluate the progress (see supplemental table for the list of items in the program).

Lessons or motivational messages are pushed every day on the dashboard of the patients, alternatively in diet, physical activity, well-being, and motivation topics. The sending of the lessons is individualized depending of the patient’s online assessments. For instance, a patient with a high cognitive restriction or high emotional eating score first receive advices on eating behavior regulation, to improve own feelings of hunger and satiety, and then dietary advices. A patient with a low level of physical activity first receive explanations on how physical activity can benefit to her health during pregnancy, and how daily physical activity can be easily increased, and then exercise advices.

A repeat evaluation at 32 weeks (± 3 weeks) using the self-administered questionnaires help personalize the second part of lessons of the e-learning program. A final evaluation is performed during the post-partum, up to the 10th week of post-partum, to determine whether the intervention leads mothers to sustain healthy changes in dietary and physical activity behaviors.

#### Health care provider’s module

The professional modules help physicians remotely follow patient’s activities. Professional dashboard includes patient’s details (i.e. inclusion date, validation of profile, completion of questionnaires, self-monitoring of weight, expected date of delivery) and activity of connection (last connection date, progress in the e-learning program). These modules also include administration of the forum and edition of newsletters.

#### Social network

The platform proposes also various social networking possibilities. Using a pseudo, participants are able to connect with other participants and with the caregivers, through a secured instant message system, and a forum. Weekly sessions of 2 h-discussion are animated on the forum by each caregiver of the team alternatively, to stimulate the interactivity and to answer the questions on all topics of the program (motivation, well-being, nutrition, eating behaviors, physical activity, pregnancy and breastfeeding).

#### Enhancing adherence and compliance

Adherence to the intervention is enhanced through several manners. Motivational reminders are repeatedly sent by email every 3 days until the registration, every 3 days for 3 weeks until the completion of online questionnaires, every 10 days in absence of connection to the program. Participants are encouraged to self-monitor their weight. Newsletters including general information on the trial, educational messages, and seasonal topics are sent to the participants by e-mail every month and posted on the platform. Finally, the forum offers an opened discussion room to participants and caregivers in order to promote interaction. Compliance to the program is assessed by evaluating the time spent on the platform and the percentage of completeness of the e-learning program. Acceptance of the program will be assessed in the intervention group, by monthly satisfactory questionnaires.

#### Participant timeline

The intervention takes place from 22 weeks of pregnancy throughout the pregnancy and 10 weeks of post-partum. The program starts after completion of the initial online questionnaires. It is composed by 3 sessions of 10 weeks, i.e. 20 weeks during pregnancy and 10 weeks in post-partum. Finally, the participation lasts 8 months after randomization of a given subject.

### Endpoints

The primary endpoint consists of the rate of obstetrical interventions during labor and delivery, defined as caesarean section and instrumental delivery (forceps and vacuum extractor). As secondary endpoints, we include data routinely collected as part of usual antenatal and perinatal care, such as GWG (measured with a scale at each visit), gestational hypertension (defined as systolic blood pressure ≥ 140 mmHg and/or diastolic blood pressure ≥ 90 mmHg after 20 weeks), preeclampsia (defined as gestational hypertension and proteinuria ≥0.30 g/24 h), GDM (diagnosed by a 75 g-oral glucose tolerance test between 24 and 28 weeks according to the guidelines of the International Association of the Diabetes and Pregnancy Study Groups (IADPSG)). Fetal and neonatal collected outcomes, consist of premature birth (defined as birth before 37 weeks), gestational age at birth, birth weight, rate of macrosomia (defined as birth weight > 90th percentile for gestational age), Apgar score, arterial umbilical cord pH, neonatal traumatism, hyperbilirubinemia, respiratory distress syndrome (i.e. needs for oxygen) and transfer in neonatal intensive care unit. Post-natal outcomes are duration of breastfeeding, maternal weight retention and child weight at the postnatal visit (8 ± 2 weeks’ post-partum).

### Sample size

As mentioned, the main objective of this study is to demonstrate the superiority of an electronic-personalized program over standard care to reduce the rate of delivery interventions (defined as caesarean section and instrumental (vacuum extractor and forceps) extractions) in obese pregnant women. In the AUDIPOG database of the French maternities, the rate of delivery interventions is 50% among obese pregnant women. We assumed that our electronic-personalized program could reduce this rate to 40% (i.e. a relative risk reduction of 20%). To detect this difference, using a 2-sided Chi-square test with an alpha risk of 5% and a power of 80%, we calculated that a total of 774 subjects (i.e. 387 subjects in each group) would be required (computation made using the PASS 12). Considering a maximum of 10% of drop outs or missing data, we will recruit 860 patients.

### Data collection

#### Clinical variables

At inclusion visit between 12 and 22 weeks, demography, medical and family history and current pregnancy health information are collected. Anthropometric variables (weight and height) are measured; blood and urine samples are drawn. No additional follow-up visit is required, as women benefit of the usual care for pregnancy (according to the French guidelines) which consists of 7 prenatal visits and 1 post-natal visit between 6 and 10 weeks after delivery. Information is collected from maternal medical records regarding health during pregnancy including gestational hypertension, preeclampsia and GDM. All women in both groups undergo a 75 g-oral glucose tolerance test (75 g glucose oral load after a fasting period of 10 h minimum) between 24 and 28 weeks. The diagnosis of GDM is made according to the IADPSG criteria (i.e. fasting plasma glucose ≥5.1 mmol/L [0.92 g/L] and/or 1-h glucose ≥10 mmol/L [1.80 g/L] and/or 2-h glucose ≥8.5 mmol/L [1.53 g/L]). The beginning of labor and the delivery mode are collected as well as the occurrence of post-partum hemorrhage. Neonatal and postnatal outcomes include the occurrence of traumatism due to childbirth, Apgar score, umbilical cord pH, admission to neonatal unit and use of phototherapy for hyperbilirubinemia. To address the influence of the intervention on neonatal growth and adiposity, neonatal anthropometric variables (weight, length, adiposity skinfolds) are measured within the first 24 h. At the post-natal visit, maternal demographic data, health since the delivery (post-partum complications such as fever, hemorrhage, thromboembolic events) and duration of breastfeeding are obtained. Maternal anthropometric variables (weight, height) are measured. To address the safety and the impact of the intervention on child health, details regarding the health since birth is also obtained. To address the influence of the intervention on infant adiposity, infant length and weight are measured at birth.

#### Online assessments

Information on diet habits, eating behaviors, physical activity, motivation stage is collected through self-administered validated questionnaires. The Short Dietary Questionnaire (SDQ) estimates energy nutrient and food intakes based on 14 items of food frequency consumption [32]. The Three Factors Eating Questionnaire (TFEQ) [33] is a food intake-behavior related questionnaire, which contains 21 items and measures three dimensions of eating behavior (cognitive restraint of eating, emotional eating, and uncontrolled eating). The Pregnancy Physical Activity Questionnaire (PPAQ) [34] is a questionnaire of 36 items enable to evaluate the duration, frequency, and intensity of physical activities in pregnant women. The motivation stage is estimated by questioning on the stage of changing [35] in order to follow a healthier diet and practice more physical activity. It contains two 5 stage-questions (see supplemental material). Questionnaires are filled three times during the trial, at the inclusion (between 22 and 25 weeks), at the mid-course of the intervention (between 32 and 35 weeks) and after delivery (up to 10 weeks’ post-partum).

### Data management

The data collected along the visits are entered through an electronic case report form (eCRF) using Clinsight software. The data of the participants collected from the web-based platform (through the self-administered questionnaires) are protected by a secured access. The data are stored in a secured server provided by Bepatient™.

### Statistics

Statistical analyses will be independently performed by the Biostatistics Department of University of Lille. Data will be analyzed using the SAS software (SAS Institute Inc., Cary, NC, USA) and all statistical tests will be performed with a 2-tailed alpha risk of 0.05. A detailed statistical analysis plan will be written and finalized prior to the database lock. For each exploratory analysis, a statistical analysis plans will be provided before any data analysis. Baseline characteristics will be described for each group. Quantitative variables will be expressed as mean (± standard deviation), median (± interquartile range) and 95% confident interval. Qualitative variables will be expressed as frequencies and percentages. Normality of distribution will be assessed graphically and using the Shapiro-Wilk test. The primary analysis for primary and secondary endpoints will be conducted on the intention-to-treat principle. A secondary per-protocol analysis will be performed.

The primary endpoint (rate of delivery interventions) will be compared between the two groups using the Chi-square test. Absolute and relative risk difference (intervention vs. control) and their 95% confidence intervals will be calculated. For the secondary objectives, binary endpoints will be compared between the two groups using chi-square tests or Fisher’s exact tests when the expected cell frequency will be inferior to 5. Quantitative endpoints will be compared between the two groups using student *t* tests or Mann-Whitney U tests in case of non-normal distribution (except if a log-transformation can be applied to normalize the data). For quantitative endpoints involving repeated measures, a linear mixed model will be used. This model allows to perform an ANOVA for repeated measures taking into account the correlation between the repeated measures. The choice of the covariance in the models will be based on the AIC criteria and normality of residuals will be checked.

## Discussion

In our Randomized Control Trial, we aim to demonstrate that an e-personalized intervention focused on nutritional counselling and adequate physical activity in pregnant obese women leads to a reduction in delivery procedures (i.e. reduced risk of caesarean section and instrumental deliveries). This program also aims to reduce the risk of maternal and neonatal complications by allowing a better control of GWG. This approach would contribute to maintain or improve the health capital of the mothers and their newborns in the long term. In terms of public health, this action offers, as a primary care, an inexpensive solution that could be proposed to a large number of obese pregnant women. We aim to show that this intervention included in the daily lives of obese pregnant women with reasonable and pragmatic objectives can be associated with a better adherence while a similar effectiveness is maintained in comparison with more complex programs. Our most important challenge is to create for participants a mind-set that motivates them to change their lifestyle, in other words to help them understand how eating healthy food and being active in pregnancy is important for their health and the health of their offspring. This program will also give the tools and supports which they need to reach their objectives. ePPOP-ID will give them self-confidence and self-esteem necessary to instill positive self-images.

Pregnancy is a very privileged time to have or to keep good health habits and to diffuse them within the family to promote more sustainable lifestyle improvements. Time of pregnancy seems to be a privileged moment during which future parents pay more attention to establish lifestyle changes. This project will offer to the health institutions and to stakeholders’ actions to be disseminated widely in pregnant obese patients and their caregivers. The broader goal is to contribute to the development of therapeutic and preventive tools to limit the dramatic consequences of obesity in pregnancy and to fight against the growing obesity epidemic by acting at a key moment of the women life.

## Supplementary information

**Additional file 1: Table S1.** Content of the e-learning program. Table giving more detailed information about the program

**Additional file 2: Method S1.** The Stages of change Questionnaire. Questionnaire created to evaluate the woman’ stage of change

## Data Availability

Not applicable.

## Abbreviations

BMI: Body Mass Index
GDM: Gestational Diabetes Mellitus
GWG: Gestational weight gain
IOM: Institute of medicine
LGA: Large for Gestational Age
PPAQ: Pregnancy Physical Activity Questionnaire
SGA: Small-for-gestational-age
TFEQ: Three Factors Eating Questionnaire
