# Supplementary material for: Rationale and design of ePPOP-ID: a multicenter randomized controlled trial using an electronic-personalized program for obesity in pregnancy to improve delivery
Source: BMC Pregnancy Childbirth. 2020 Oct 7;20:602. doi: 10.1186/s12884-020-03288-x (PMC7542973; doi:10.1186/s12884-020-03288-x)
Supplement: Supplementary file 1 — Additional file 1: Table S1. Content of the e-learning program. Table giving more detailed information about the program [file 12884_2020_3288_MOESM1_ESM.docx]

**Table S1: Content of the e-learning program**

| **TOPIC** | **FIELD** | **TITLE** |
| --- | --- | --- |
| **HEALTHIER DIET** "Good to know" | My weight evolution in pregnancy | Benefits of appropriate gestational weight gain for my baby and me |
|  | My weight evolution in pregnancy | Natural weight evolution in pregnancy |
|  | My weight evolution in pregnancy | Happy Quiz : do I have to eat for two in pregnancy ? |
|  | Food at risk | Listeria and toxoplasmosis infection: how to reduce the risk ? |
|  | Toxic substances to avoid in pregnancy | Alcohol, tobacco and drugs |
|  | Toxic substances to avoid in pregnancy | Coffee and sweet drinks : do I have to avoid them too ? |
|  | Vitamins in pregnancy | Why (and when) do I have to take iron, folate, vitamin D and iodine ? |
|  | Vitamins in pregnancy | Happy Quiz about vitamins |
|  | Optional lesson | Gestational diabetes : how to manage it ? |
|  | Optional lesson | Vegan diet, gluten free, … : how to manage ? |
|  | Optional lesson | Food allergies : how to manage ? |
|  | Optional lesson | Eat more fruits and vegetables : how to succeed ? |
| **HEALTHIER DIET** "In practice" | Recommended diet in pregnancy | I eat tasty and healthy |
|  | How can I improve my diet in practice ? | When do I eat ? (managing hunger) |
|  | How can I improve my diet in practice ? | How do I recognize satiety ? |
|  | How can I improve my diet in practice ? | What do I put in my plate ? |
|  | How can I improve my diet in practice ? | Cravings : how can I manage ? |
|  | How can I improve my diet in practice ? | Palatable food, sweet, fatty and salty : allowed or not ? |
|  | How can I improve my diet in practice ? | Needs evolving along pregnancy term |
|  | How can I improve my diet in practice ? | To understand everything on nutritional labels |
| **HEALTHIER DIET** "Idea for recipes" | Cooking idea to delight | Fall and winter menus |
|  | Cooking idea to delight | Spring menus |
|  | Cooking idea to delight | Summer menus |
| **HEALTHIER DIET** "Breastfeeding" | Breastfeeding | Why breastfeed ? |
|  | Breastfeeding | Advice for breastfeeding |
|  | Breastfeeding | Advice for breastfeeding, in practice |
|  | Breastfeeding | And after breastfeeding ? |
|  | Breastfeeding | Recommended diet during breastfeeding |
|  | Breastfeeding | Toxic substances to avoid during breastfeeding |
|  | Breastfeeding | Vitamins : do we have to take any ? |
|  | Breastfeeding | Happy Quiz on breastfeeding |
| **MORE PHYSICAL ACTIVITY** "Good to know" | Physical activity in pregnancy | What is physical activity ? And why work out in pregnancy ? |
|  | Physical activity in pregnancy | Benefits of physical activity in pregnancy |
|  | Physical activity in pregnancy | Effects of abs on my body |
|  | Physical activity in pregnancy | What are aerobic activities ? |
|  | Physical activity in pregnancy | What is muscle strengthening ? |
|  | Physical activity in pregnancy | What is stretching ? |
|  | Physical activity in pregnancy | How can pregnancy affect physical activity ? |
|  | Soft gym in pregnancy | Benefits of soft gym in pregnancy |
|  | Measuring physical activity with new technologies | Why is it important to monitor physical activity ? |
| **MORE PHYSICAL ACTIVITY** "Warnings" | Recommended physical activity for pregnant women | Physical activity recommendations during pregnancy |
|  | Recommended physical activity for pregnant women | Why is it important to drink water during physical activity ? |
|  | Recommended physical activity for pregnant women | What are the precautions to take when practicing physical activity ? |
|  | Physical activity during pregnancy | Which physical activities during pregnancy ? |
|  | Physical activity during pregnancy | Which physical activities require precautions ? |
|  | Physical activity during pregnancy | Which physical activities to avoid during pregnancy ? |
| **MORE PHYSICAL ACTIVITY** "Aquagym program" | Swimming during pregnancy | Exercises to strengthen muscles |
|  | Swimming during pregnancy | Stretching exercises |
| **MORE PHYSICAL ACTIVITY** "Gym program" | Soft gym during pregnancy | Soft gym benefits during pregnancy |
|  | Soft gym during pregnancy | Basic postures |
|  | Soft gym during pregnancy | Home exercises : warming up |
|  | Soft gym during pregnancy | Home exercises: upper body muscles strengthening |
|  | Soft gym during pregnancy | Home exercises: lower body muscles strengthening |
|  | Soft gym during pregnancy | Home exercises: stretching |
| **MORE PHYSICAL ACTIVITY** "In practice" | Monitoring physical activity with new technologies | What are the new devices to monitor my physical activity ? |
|  | Improving physical activity | How can I improve my physical activity |
|  | Improving physical activity | Sport associations near me |
|  | And after pregnancy ? | Physical activity after birth |
| **TO BE MOTIVATED** "To change" | I equip myself to change | Is it mandatory to change ? |
|  | I equip myself to change | A state of mind |
|  | I equip myself to change | To prepare myself |
|  | I equip myself to change | Vision-engagement |
|  | I equip myself to change | Self-coaching. |
|  | I equip myself to change | Changing is a question of objectives : basis |
|  | I equip myself to change | Changing is a question of objectives : objectives process |
|  | I equip myself to change | Changing is a question of objectives : alive process |
|  | I equip myself to change | Changing is a question of objectives : boosted objectives |
|  | I equip myself to change | Motivation sources to succeed |
| **TO BE MOTIVATED** "To feel well" | Towards healthier lifestyles | Why is it important to feel well to change ? |
|  | Towards healthier lifestyles | I refine my well-being: I enjoy ! |
|  | Towards healthier lifestyles | I refine my well-being: I stay in the present time, here and now ! |
|  | Towards healthier lifestyles | I take consciousness of the importance of others in my life |
|  | Towards healthier lifestyles | I develop my emotional skills : I identify my emotions |
|  | Towards healthier lifestyles | I develop my emotional skills : I understand my emotions and the ones of others |
|  | Towards healthier lifestyles | I develop my emotional skills : I express my emotions |
|  | Towards healthier lifestyles | I develop my emotional skills : I regulate my emotions |
|  | Towards healthier lifestyles | I develop my emotional skills : I regulate my negative emotions |
| **TO BE MOTIVATED** "In case of failure" | I do not feel motivated | I do not see the point in changing anything, I already feel very good |
|  | I do not feel motivated | Diet and exercises are not my priority at the present time |
|  | I do not feel motivated | Healthier diet, more exercises ? I already I will not succeed |
|  | I do not feel motivated | I am motivated but I do not succeed |
|  | I do not feel motivated | I do not feel very well, I will keep an eye on my thoughts |
|  | I do not feel motivated | I criticize myself ? I keep an eye on my thoughts |
|  | I do not feel motivated | Tiredness, blues, fed up, I do not want to do any more effort. |
|  | I do not feel motivated | Sometimes, I loose control |
| **WELL-BEING** "Pregnancy without stress" | My pregnancy without stress | I enjoy my pregnancy |
|  | My pregnancy without stress | I avoid stress |
| **WELL-BEING** "Sophrology" | Sophrology in pregnancy | Benefits of relaxation during pregnancy |
|  | Sophrology in pregnancy | Relaxation practice |
| **WELL-BEING** "Yoga" | Yoga practice in pregnancy | Benefits of prenatal yoga |
|  | Yoga practice in pregnancy | Practicing yoga nest: advice before starting |
|  | Yoga practice in pregnancy | Lower body exercises |
|  | Yoga practice in pregnancy | Exercises to open the pelvis |
|  | Yoga practice in pregnancy | Exercises for perineum |
|  | Yoga practice in pregnancy | Exercises for back pain relief |
|  | Yoga practice in pregnancy | Exercises for relaxation |
|  | Yoga practice in pregnancy | Exercises for energy |
|  | Yoga practice in pregnancy | Exercises for balance |
|  | Yoga practice in pregnancy | Exercises to prepare delivery |
|  | Yoga practice in pregnancy | Yoga postures slideshow |
