# Supplementary material for: Rationale and design of ePPOP-ID: a multicenter randomized controlled trial using an electronic-personalized program for obesity in pregnancy to improve delivery
Source: BMC Pregnancy Childbirth. 2020 Oct 7;20:602. doi: 10.1186/s12884-020-03288-x (PMC7542973; doi:10.1186/s12884-020-03288-x)
Supplement: Supplementary file 2 — Additional file 2: Method S1. The Stages of change Questionnaire. Questionnaire created to evaluate the woman’ stage of change [file 12884_2020_3288_MOESM2_ESM.docx]

**Method S1: The Stages of change Questionnaire**

**About improving your current diet, please sincerely check only one box:**

I do not have any intention to change

I may change in a few months, I do not know yet

I am preparing to change my diet by the next month

I started to change my diet the 6 last months

I have changed my diet for more than 6 months

**About improving your current physical activity, please sincerely check only one box:**

I do not have any intention to change

I may change in a few months, I do not know yet

I am preparing to change my physical activity by the next month

I started to change my physical activity the 6 last months

I have changed my physical activity for more than 6 months
